# Salivary Calculus

**Published:** 1887-08

**Authors:** 


### Salivary Calculus.

Polak reports the case of a tumor developing in the jaw of an otherwise sound man, 31 years old, subsequent to the extraction of a carious tooth. It extended from the border of the lower jaw to the thyroid cartilage and has a fistulous opening at the anterior border of the sternocleido-mastoid through which pus escaped. Various applications had no effect. An injection of iodine solution escaped into the mouth through the caruncula sublingualis. Since it appeared to be a tumor of the sublingual gland, a part was excised, and eleven days later a further excision was made, but there was no improvement. In sounding the opening twelve days after this, a rough, hard body was felt in the bottom that seemed to be the necrosed jaw bone. Upon the next day, when the resection of the jaw was attempted, a salivary calculus weighing 23 grains was found. It had been raised during the night from the bottom of the abscess to the surface. The healing of the parts at once proceeded without interruption. —*Centralb l. f. d. med. Wissensch, May* 21, 1887.

